# Supplementary material for: Polyketide synthase gene clusters in the ericoid mycorrhizal fungus Oidiodendron maius: genome-wide prediction, expression profiling, and functional characterization of knockout mutants
Source: Front Plant Sci. 2026 Sep 16;17:1924430. doi: 10.3389/fpls.2026.1924430 (PMC13623903; doi:10.3389/fpls.2026.1924430)
Supplement: Supplementary file 1 [file SupplementaryFile1.zip › Supplementary Materials/Supplementary Folder 1.zip!/Oidma1_AssemblyScaffolds_Repeatmasked/knownclusterblast/cluster19/jgi.p_Oidma1_41487_mibig_hits.html]

| MIBiG Protein | Description | MIBiG Cluster | MiBiG Product | % ID | % Coverage | BLAST Score | E-value |
| --- | --- | --- | --- | --- | --- | --- | --- |
| AFT91402.1 | hypothetical\_protein | BGC0000338\_c2 | Echinocandin\_B\_biosynthetic\_gene\_cluster | 47 | 97.7911646586 | 456 | 4.8e-128 |
| AFY23020.1 | betaine\_aldehyde\_dehydrogenase | BGC0000274 | Tetarimycin\_biosynthetic\_gene\_cluster | 47 | 93.9759036145 | 409 | 6.8e-114 |
| ABW71833.1 | putative\_aldehyde\_dehydrogease | BGC0000303 | Anthramycin\_biosynthetic\_gene\_cluster | 36 | 95.983935743 | 297 | 2.9e-80 |
| ABC36740.1 | aldehyde\_dehydrogenase | BGC0001120 | Burkholderic\_acid\_biosynthetic\_gene\_cluster | 37 | 95.1807228916 | 280 | 3.6e-75 |
| ABC35115.1 | aldehyde\_dehydrogenase\_family\_protein | BGC0001102 | Malleilactone\_biosynthetic\_gene\_cluster | 36 | 92.9718875502 | 268 | 1.9e-71 |
| AAS47555.1 | putative\_aldehyde\_dehydrogenase | BGC0001108 | Pederin\_biosynthetic\_gene\_cluster | 35 | 94.9799196787 | 267 | 2.4e-71 |
| ABB90277.1 | aldehyde\_dehydrogenase | BGC0001057 | Zearalenone\_biosynthetic\_gene\_cluster | 35 | 93.1726907631 | 260 | 5.1e-69 |
| AFL48539.1 | putative\_succinic\_semialdehyde\_dehydrogenase | BGC0000084 | Laidlomycin\_biosynthetic\_gene\_cluster | 34 | 92.1686746988 | 259 | 6.7e-69 |
| CAJ14050.1 | putative\_aldehyde\_dehydrogenase | BGC0000406 | Phosphinothricin\_biosynthetic\_gene\_cluster | 36 | 94.9799196787 | 245 | 1.7e-64 |
| WP\_030845402.1 | aldehyde\_dehydrogenase | BGC0001368 | Tambromycin\_biosynthetic\_gene\_cluster | 34 | 94.1767068273 | 239 | 9.3e-63 |
| ABY65994.1 | aldehyde\_dehydrogenase | BGC0001008 | Maduropeptin\_biosynthetic\_gene\_cluster | 33 | 92.7710843373 | 222 | 9e-58 |
| CAE45692.1 | hypothetical\_protein | BGC0000031 | Borrelidin\_biosynthetic\_gene\_cluster | 32 | 91.3654618474 | 218 | 1.3e-56 |
| AAD32735.1 | MmcL | BGC0000915 | Mitomycin\_biosynthetic\_gene\_cluster | 34 | 91.3654618474 | 213 | 5.5e-55 |
| AAW03334.1 | hypothetical\_protein | BGC0000982 | Cystothiazole\_A\_biosynthetic\_gene\_cluster | 30 | 91.9678714859 | 195 | 1.5e-49 |
| ACI30658.1 | methylmalonate\_semialdehyde\_dehydrogenase | BGC0000313 | Beauvericin\_biosynthetic\_gene\_cluster | 27 | 95.7831325301 | 191 | 2.9e-48 |
